# A Probabilistic Model for Hydrokinetic Turbine Collision Risks: Exploring Impacts on Fish

**DOI:** 10.1371/journal.pone.0117756

**Published:** 2015-03-02

**Authors:** Linus Hammar, Linda Eggertsen, Sandra Andersson, Jimmy Ehnberg, Rickard Arvidsson, Martin Gullström, Sverker Molander

**Affiliations:** 1 Chalmers University of Technology, Department of Energy and Environment, Gothenburg, Sweden; 2 Stockholm University, Department of Ecology, Environment and Plant Sciences, Stockholm, Sweden; 3 Marine Monitoring AB, Lysekil, Sweden; Pacific Northwest National Laboratory, UNITED STATES

## Abstract

A variety of hydrokinetic turbines are currently under development for power generation in rivers, tidal straits and ocean currents. Because some of these turbines are large, with rapidly moving rotor blades, the risk of collision with aquatic animals has been brought to attention. The behavior and fate of animals that approach such large hydrokinetic turbines have not yet been monitored at any detail. In this paper, we conduct a synthesis of the current knowledge and understanding of hydrokinetic turbine collision risks. The outcome is a generic fault tree based probabilistic model suitable for estimating population-level ecological risks. New video-based data on fish behavior in strong currents are provided and models describing fish avoidance behaviors are presented. The findings indicate low risk for small-sized fish. However, at large turbines (≥5 m), bigger fish seem to have high probability of collision, mostly because rotor detection and avoidance is difficult in low visibility. Risks can therefore be substantial for vulnerable populations of large-sized fish, which thrive in strong currents. The suggested collision risk model can be applied to different turbine designs and at a variety of locations as basis for case-specific risk assessments. The structure of the model facilitates successive model validation, refinement and application to other organism groups such as marine mammals.

## Introduction

Recent years have seen a growing development of tidal power, including the deployment of several pre-commercial units in different parts of the world [1–3]. Modern tidal power mostly concerns hydrokinetic turbines which generate electricity from the kinetic energy of fast-flowing water. Some of the now operating turbines have large rotors sweeping through the water with blade-tip velocities exceeding 10 ms^−1^. Because most marine animals move at considerably lower velocities, concerns have been raised regarding potential collisions between hydrokinetic turbines and fish, marine mammals and diving birds [4–7]. Should hydrokinetic turbines cause high mortality to some species, this may have substantial impact on vulnerable populations.

Few field observations of interactions between marine animals and hydrokinetic turbines have hitherto been reported. Quantitative data are only available from two studies on fish around small vertical-axis turbines [8,9] and one study on fish at a ducted turbine [10]. The studies by Broadhurst and Barr [10] and Hammar *et al*. [9] were limited to daytime conditions, and no collisions were observed. The study by Viehman [8], however, recorded a higher number of small pelagic fish entering the rotor during night compared to daytime, although collisions could not be verified. Regarding large-diameter open-flow hydrokinetic turbines, only occasional observations from a 5 m diameter turbine are available [11], showing that interactions with fish occur.

In lack of conclusive data, potential impacts of large hydrokinetic turbines have been explored through collision risk modeling [12–17]. These models relate, more or less, to established and rather accurate conventional hydropower blade-strike models [18–20]. However, hydropower systems essentially differ from hydrokinetic turbines; most importantly because fish are entrained through the system with little ability to avoid the turbine.

The different hydrokinetic turbine models have some core similarities but differ in extent of scope (coverage of the collision risk pathway) and in consideration of animal behavior. There is consensus that animal behavior is important with respect to collision risks, but methods of incorporating behavior in models differ. For instance, it is generally assumed that natural animal movement patterns will influence the exposure to turbines in the first place. Here, the probability of being at the same depth and location as a turbine has often been considered [11,13,14,16], but rarely with emphasis on the particular behavior in strong currents. It is further expected that avoidance capability affects the outcome of animal–turbine interactions. In previous models, this avoidance has been addressed either as burst swimming towards the turbine [14] or as assigned avoidance probabilities [16]. None of the previous studies have modeled avoidance behavior in detail and some have, as a precaution due to uncertainties, decided not to include avoidance [11,13,17]. The only collision risk model published in the scientific literature takes a thorough approach to fluid dynamics but does not involve fish behavior [17]. This fragmented and incoherent situation among existing collision risk models and their shortcomings hampers comprehensive risk assessment and risk management, calling for a more inclusive model.

In this study, we aim to make a synthesis of previous model works into a generic collision risk model in which different model components are explicitly separated and behavioral traits are included. We explore the synthesis model using fish and large open-flow tidal turbines as examples. We focus particularly on two of the behavior-related model components, where very little information has been reported [21,22], providing novel data from a video-based study of fish movements in strong tidal currents and developing a model for simulating fish avoidance behavior. Based on the findings of the implemented model, we discuss apparent ecological risks and risk-reducing technical measures.

The presented collision risk model is based on fault tree analysis, which is a method used within the field of probabilistic risk assessment [23]. A fault tree diagram explicitly shows all different relationships between events that are necessary for a so-called top event to occur. By assigning probabilities (generic or case-specific) to each event, the probability of *turbine mortality* can be quantified. As research progresses, the general collision risk model presented in this paper can be further developed by incorporating additional and more detailed branches in the fault tree.

## The Suggested Collision Risk Model

To understand the ecological risk caused by hydrokinetic turbines, the number of specimens from a specific population that may be lost due to turbines should be related to the population size, and factors influencing population dynamics. At the basic level, population size (*N*_*TOT*_) is a function of the number of births (*N*_*B*_), deaths (*N*_*D*_), immigrants (*N*_*I*_) and emigrants (*N*_*E*_) over time [24]. The number of deaths may include both natural mortality (from e.g. predation) and mortality caused by human activities (e.g. fisheries). In this case, the number of deaths caused by hydrokinetic turbines (*N*_*TM*_) per time can be added to the function, as illustrated in **Eq. 1**.

$$\frac{dN_{TOT}}{dt}=\frac{dN_{B}}{dt}+\frac{dN_{I}}{dt}-\frac{dN_{D}}{dt}-\frac{dN_{E}}{dt}-\frac{dN_{TM}}{dt}$$ (Eq. 1)

If *N*_*TM*_ affects *N*_*TOT*_ to the extent that it lowers the population viability over time, then the turbines pose a significant risk to the population. Population viability can be understood as the survival of a population in a state that maintains its vigor and its potential for evolutionary adaptation [25]. So by use of **Eq. 1**, the collision risk from turbines can be related to the total population size in an ecologically relevant manner.

**Fig 1:**
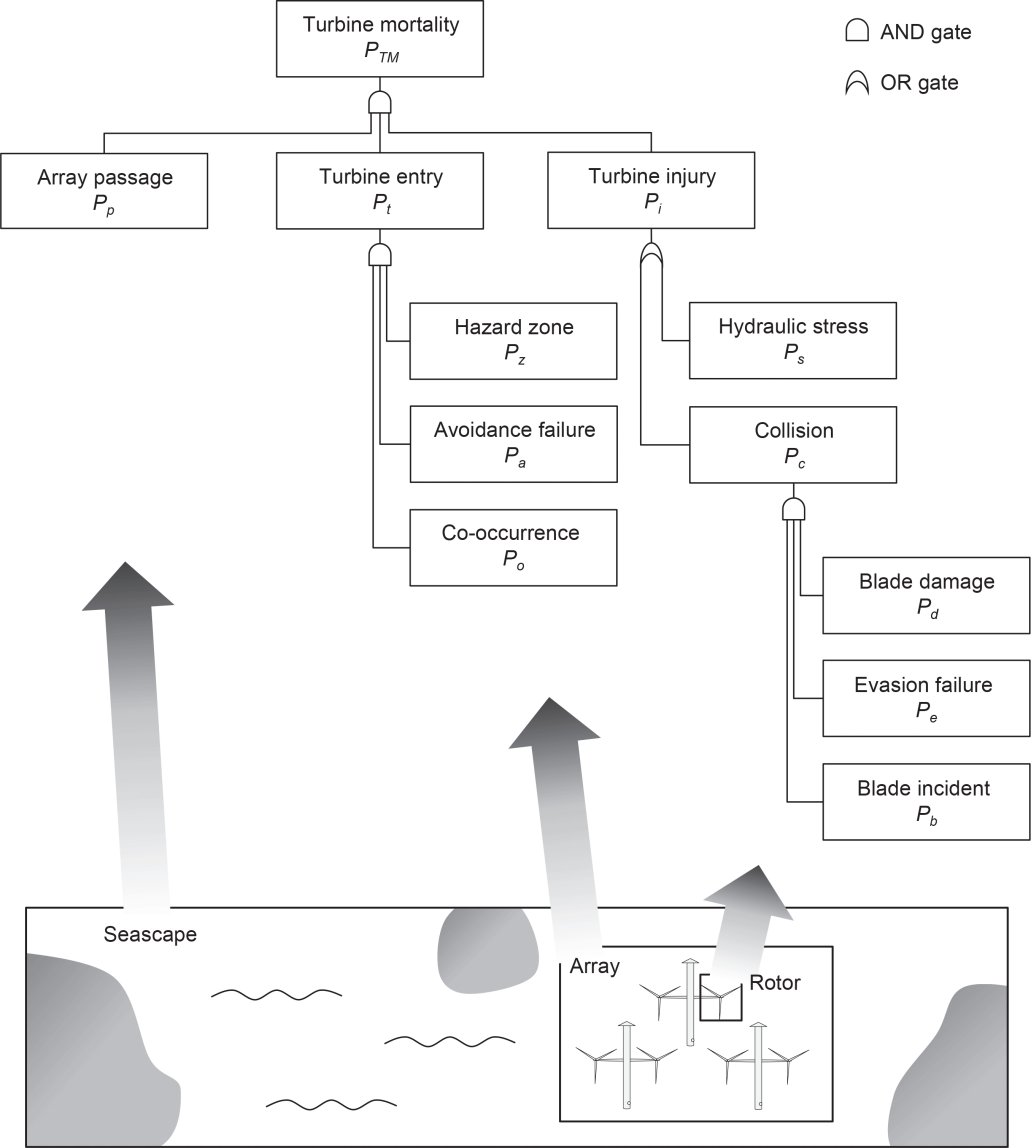
The generic collision risk model described as a fault tree diagram. Turbine mortality is the top event. Intermediate events are connected to basic events through AND gates and OR gates. The probability of each basic event is case-specific and should be assigned using applied equations or estimates. If detailed data on fish movements within the array are available *P*_*o*_ will be accurate and *P*_*p*_ can be removed (see section on *P*_*o*_). The lower panel indicates how the *P*_*p*_, *P*_*t*_ and *P*_*i*_ deals with events at the scale of population range, at the scale of the array and turbines and at the scale of the rotor blades, respectively. Each basic event, referred to as model components, is examined in the main text and an example of model implementation is provided.

Based on previous discussions on hydrokinetic turbine collision risks [13,14,22], the following events are required for collision related mortality to occur: (1) the turbine and the animal must be in the same place at the same time, (2) the animal must fail to avoid the hazardous part of the turbine and (3) the animal must fail to pass safely through the rotor swept disc. We conceptualize this chain of events as the fault tree based collision risk model illustrates in **Fig. 1**. Fault trees are based on Boolean logic (using operations such as AND and OR) and all events are binary; they have an assigned probability of occurring or not occurring [23]. For stochastic modelling, probability distributions can be used.

The top event, turbine mortality (*P*_*TM*_), is the probability of suffering severe injury from turbines for a given specimen in a given time interval. By using population-representative values for the biological input parameters, *P*_*TM*_ can be multiplied by *N*_*TOT*_ to give *N*_*TM*_ for the specified time interval. Since many underlying parameters vary over time, so does the probability of turbine mortality. *P*_*TM*_ and *N*_*TM*_ should therefore be calculated for a number of different settings (note that, in theory, *N*_*TOT*_ also varies with time). A first step when implementing the model is therefore to select a suitable time interval (e.g. one hour) and a few representative settings as a basis for a *yearly N*_*TM*_, which covers the existing site-specific variations in the specific probabilities (see **Table 1**).

**Table 1:** Example for calculating *yearly N*_*TM*_ based on settings A-H.

**Setting for calculating *hourly N*_*TM*_**	***hourly N_TM_***	**Hours**	***yearly N_TM_***
A Ebb/flood, daytime, season 1	*hourly N_TM_A*	1643	*hourly N*_*TM*_A × 1643
B Slack, daytime, season 1	*hourly N_TM_B*	547	*hourly N*_*TM*_B × 547
C Ebb/flood, night, season 1	*hourly N_TM_C*	1643	*hourly N*_*TM*_C × 1643
D Slack, night, season 1	*hourly N_TM_D*	547	*hourly N*_*TM*_D × 547
E Ebb/flood, daytime, season 2	*hourly N_TM_E*	1643	*hourly N*_*TM*_E × 1643
F Slack, daytime, season 2	*hourly N_TM_F*	547	*hourly N*_*TM*_F × 547
G Ebb/flood, night, season 2	*hourly N_TM_G*	1643	*hourly N*_*TM*_G × 1643
H Slack, night, season 2	*hourly N_TM_H*	547	*hourly N*_*TM*_H × 547
*Yearly total*		*8760*	*SUM*

The *hourly N*_*TM*_ values are here calculated for different settings (A-H) representing diurnal or semidiurnal tides and two different, equally long, seasons. The *yearly N*_*TM*_ is then given as the sum of *hourly N*_*TM*_ multiplied by number of hours.

A first prerequisite for turbine mortality is that specimens of the investigated population pass through the area where turbines are installed. This is represented by the basic event *array passage* (*P*_*p*_), which denotes the probability that a specific specimen of the population in the time interval considered will pass through the array (area with multiple turbines). If the turbine array constitutes an important part of the population’s range, the array passage will be particularly high. The probability of specimens passing through the array to perish as a cause of a turbine in turn depends on what happens inside the array. First, the specimen has to come across a turbine and enters the hazardous part of the turbine (*turbine entry*, *P*_*t*_). This probability partly depends on the natural movement pattern of the animal, that is, the probability of specimens moving with the current at the same depth as the rotor blades while the turbine is in operation (*co-occurrence*, *P*_*o*_). It also depends on the probability of failing to successfully undertake an avoidance action and swim away from the turbine once it is detected (*avoidance failure*, *P*_*a*_) and on the probability of being swept by current into the hazardous zone where rotor blades move fast (*hazard zone*, *P*_*z*_). Second, turbine mortality also depends on the probability of incidents causing severe injury while the specimen passes through the hazardous part of the turbine (*turbine injury*, *P*_*i*_). This can result either from *hydraulic stress* (*P*_*s*_), such as pressure drop and shear, or *collision* (*P*_*c*_) with a rotor blade. A prerequisite for collision is that a rotor blade sweeps across the specimen’s path while it is crossing the hazardous zone, which is calculated as the probability of *blade incident* (*P*_*b*_). Furthermore, collision will only occur if the specimen fails to complete a close-range evasion (*evasion failure*, *P*_*e*_). Lastly, *blade damage* (*P*_*d*_) determines the probability of a collision to be severe. Based on this outlined event chain, and based on equations for calculation of probabilities for AND and OR gates [23], turbine mortality can be calculated as given by **Eq. 2**.

$$P_{TM}=P_{p}\times P_{t}\times P_{i}=P_{p}\times P_{o}\times P_{a}\times P_{z}\times \left(P_{s}+P_{b}\times P_{e}\times P_{d}-P_{s}\times P_{b}\times P_{e}\times P_{d}\right)\approx P_{p}\times P_{o}\times P_{a}\times P_{z}\times \left(P_{s}+P_{b}\times P_{e}\times P_{d}\right)$$ (Eq.2)

At this level of detail, the generic model is likely to be applicable for most animals and turbines. However, the probabilities for each event will differ among cases and should be calculated or estimated for each specific application of the model.

In the following sections, we go through each basic event of the collision risk model (**Fig. 1**), with a focus on collision risks for fish at large-diameter horizontal-axis tidal turbines. We use the term ‘model component’ to describe events. Two model components, regarding fish behavior in strong currents (*P*_*o*_) and fish avoidance behavior (*P*_*a*_), are examined in more detail using field data and probabilistic modeling. Lastly, the model implemented as an example.

## Array Passage

**Fig 2:**
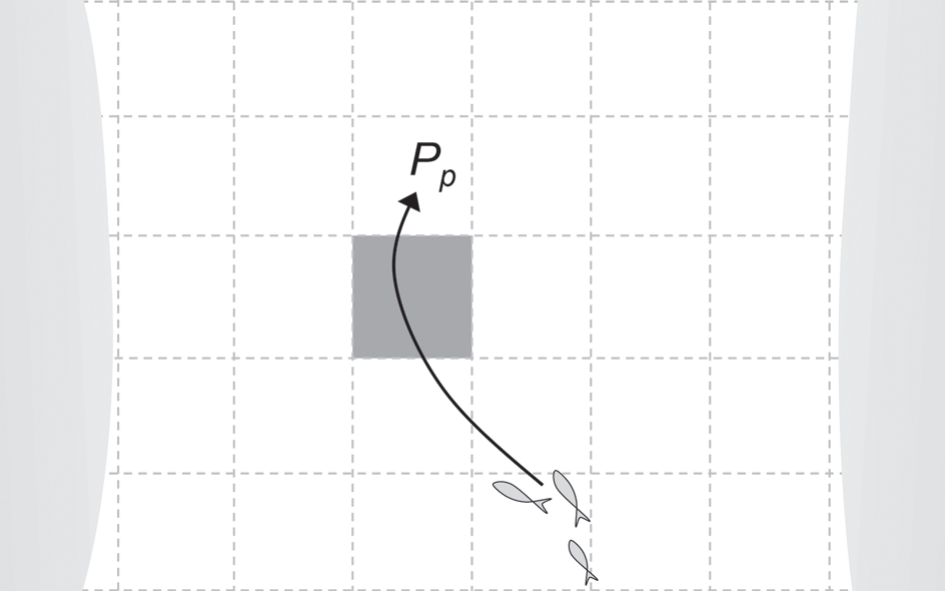
Conceptual representation of the probability of array passage (*P*_*p*_) for a given time interval. Viewed from above, the pattern square indicates a turbine array (dark square) located in an arbitrary seascape within the population’s range, consisting of 30 turbine array units (empty squares). For a given time interval a population-representative fish specimen moves 5 turbine array units, as indicated by the arrow, and *P*_*p*_ = 0.17 using **Eq. 3**.

The array passage model component (*P*_*p*_) gives the probability that a specific specimen of a given population will pass through the location of a given tidal turbine installation within a given time interval (i.e. the time interval selected for the modeling). Array passage depends on the movement pattern of the specimens in the population and on the size of the turbine array in relation to the spatial range of the population (**Fig. 2**). This can most accurately be estimated where movements through the turbine array are known, along with the population size. For instance, if 1% of the population’s specimens swim through the location of the turbine array per selected time interval, then *P*_*p*_ = 0.01.

If such site-specific data is not available, random movements and average speed can be assumed, as was done by Wilson *et al*. [13] when estimating tidal turbine collision risks for Atlantic herring and Harbour porpoise in the North Sea. One way of estimating *P*_*p*_ based on knowledge of average speed and population range is suggested by **Eq. 3**:

$$P_{p}=\frac{v_{p}}{R_{p}}$$ (Eq.3)

where *v*_*p*_ is the average swimming speed in turbine array units per time and *R*_*p*_ is the population range in turbine array units. Turbine array units represent the size of the turbine installation, as illustrated in **Fig. 2**. If the turbine array overlaps with preferred habitats such as spawning areas, foraging grounds or migration routes, an affinity factor should preferably be incorporated, increasing the probability of array passage. This approach (**Eq. 3**) involves substantial simplifications and may be most suitable for screening-type risk assessments.

## Co-occurrence

**Fig 3:**
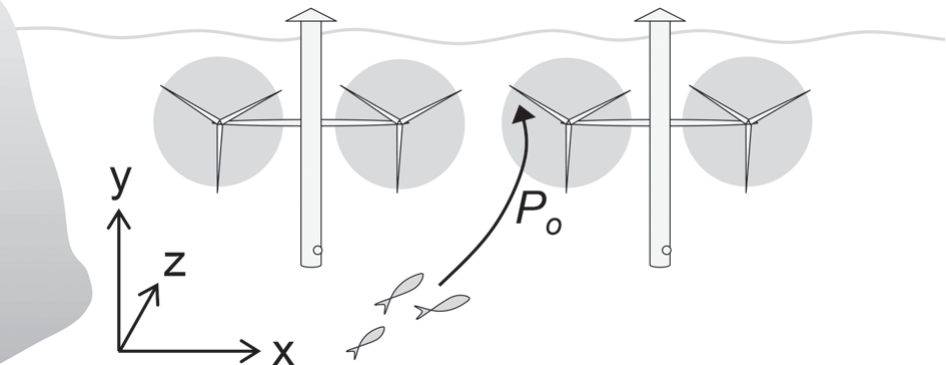
Conceptual representation of the probability of co-occurrence (*P*_*o*_) for fish passing through a turbine array. *P*_*o*_ is the probability that a given specimen that passes through an area with tidal turbines, during times when rotors are rotating, will come across a rotor. *P*_*o*_ can be described as the combined probabilities of the fish moving along current (z) at the same depth (y) and horizontal position (x) as the rotors. These probabilities are influenced by the particular behavior of fish swimming in strong currents.

Co-occurrence (*P*_*o*_) refers to the spatiotemporal overlap of a specimen and a turbine at a detailed level, inside the turbine array. The probability of co-occurrence describes the probability that passing specimens will move towards the rotor swept disc (defined by the rotor perimeter) of any of the turbines in the array, during turbine operation (**Fig. 3**). The probability of co-occurrence can be most accurately estimated by long-term site-specific field observations at turbine positions and rotor depths. By dividing the number of passing specimens per time by the total population size, an accurate measure of *P*_*o*_×*P*_*p*_ is acquired. Where such detailed data are not available, the probability of co-occurrence for specimens that pass through the array can be estimated based on understanding of fish movements in relation to strong currents.

Tidal turbines are typically positioned where currents are strongest, at some distance from the shore, for example in the middle of a strait, with rotors positioned at mid-water depth [26]. In this case, specimens moving only along the bottom or in sheltered water along the shore will not be exposed [22]. For specimens moving in the strong flows, turbine rotor diameter and array configuration, including the space between different turbines, will further influence the probability of co-occurrence [14]. As for the temporal dimension, rotors may only be hazardous while rotating, which depends on the tidal cycle. Most tidal turbines target current speeds of 2–3 ms^−1^ and have cut-in speeds around 1 ms^−1^ [1,26,27]; so at slack tide the probability of co-occurrence is zero.

To assign a probability for co-occurrence consequently requires knowledge of animal behavior with respect to current velocity. This is, however, an underdeveloped area of research because of the difficulties of quantitative biological sampling in harsh marine environments [8,22,28,29]. Water flow rates are known to influence fish distribution in riverine systems [30,31], and among marine fish taxa barracudas [32] and other predators [22] are known to thrive around current swept areas, although there is little information on more detailed current preferences. From freshwater systems it is known that benefits from high flows include increased oxygen levels and food availability [29]. In marine systems, at least increased food availability is likely to be an attractive factor of currents for many fish. But because of the high kinetic energy in flowing water, swimming against, or holding position in, a strong current is very energy demanding. Wakes behind solid structures such as rocks are therefore important micro-habitats [29,33]. In the pelagic, where there is no shelter, the influence of current speed can be expected to be particularly influential on fish movements. Stationary fish may therefore avoid the pelagic when current speeds become too high. Some non-stationary species, however, are known to undertake selective tidal stream transport as a means of migration [34,35].

One of the few studies of fish in strong currents is reported from a tidal power installation in the North Sea [10]. Here, Pollock occurred around the turbine structure at slack tide but its abundance quickly dropped when current speed increased to 0.8 ms^−1^. No fish were observed at current speeds above 1.7 ms^−1^. Another study, also related to tidal power, showed how small fish, thought to be Atlantic herring, occurred in the pelagic at speeds up to 2 ms^−1^, although densities were reported higher during slack tides [8]. In order to supplement these observations and increase the understanding of fish behavior in tidal currents, a video-based field study was carried out. Below we present the methods, findings and implications of this field study in relation to fish–turbine co-occurrence.

### Methods of the fish behavior field study

The main objective of this field study was to identify main factors describing the relationship between natural fish movements and current speed. Some additional data on fish characteristics were collected for usage in the avoidance modeling (see the subsequent section describing avoidance failure). The field observations were sampled in the Ponta Torres marine reserve, a subtropical tidal channel between Maputo Bay and the open Indian Ocean in southern Mozambique. The field work was permitted by Estação de Biologia Marítima da Inhaca, Universidade Eduardo Mondlane. The study did not involve any interaction, injury, capture or removal of animals.

The site was 8–12 m deep with sandy and rocky bottoms and high abundances of various coastal fish species [9]. The tidal currents reached 1.5 ms^−1^ at the site. Stereo-video underwater cameras were used to record fish movements in the mid-water. The camera systems were randomly shifted among three different positions where they could be safely fixed by rock outcrops and mooring lines while recording the water column. A total of 30 video samples (each 45 min) were collected. Sampling was conducted in March–April 2012 during daylight from 7 am to 5 pm.

Stereo-video systems have two cameras recording the same object from different angles so that detailed measurements of lengths and speed can be computed [9,36]. In each system, two GoPro HERO2 cameras in flat-lens underwater housings were fixed and calibrated to boards, with 0.8 m base separation and 4° convergence per camera. The SeaGIS EventMeasure software was used for analysis.

The *in situ* current speed was estimated from camera recordings of drifting debris and validated by Doppler current meter measurements [9]. Visibility (water clarity/turbidity) was estimated during the video analysis using the stereo-video measurement function [9].

Only fish taxa known to fully or partly utilize the mid- and upper parts of the water column as adults [37] were included in the study, since they are the most important in the context of collision risks [22]. Collected biological data included activity, direction, depth, speed and length of by-swimming fish. Fish activity was derived from the number of times a fish entered the recorded water volume, thus reflecting fish movements in the area. Fish activity was standardized to the entrance-area of recorded water volume in order to control for changes in visibility. This means that in water with low visibility the number of recorded fish was divided by a lower entrance-area than in waters with high visibility. Swimming direction was measured in relation to the current direction and categorized as counter-current, along-current or transverse swimming. Swimming depth was categorized as bottom- or pelagic (>2 m above bottom) swimming. Swimming speed and lengths were measured using the stereo function of the camera system.

Correlative relationships between current speed and fish activity in the pelagic were investigated through linear regression, using square root transformation to obtain homoscedasticity, or through Spearman rank correlation tests. Fish swimming at bottom (<2 m above bottom) were not included in correlative analyses. Logistic regression was used to test effects of current speed on fish swimming direction and swimming depth. Models with current speed as predictor were tested against constant models (no predictor). Logistic regression analyses were restricted to current speeds of ≥0.5 ms^−1^ in order to disclose the effect of faster currents. To ensure test validity, logistic regressions were only applied on taxa including at least 30 observed specimens in each level of the dependent variable (swimming depth: bottom vs. pelagic, swimming direction: along-current vs. counter-current).

### Results of fish behavior field study

The collected video data included 2 804 fish from 14 pelagic associated taxa with dominance of sergeants (*Abudefduf* spp.) and trevallies (*Caranx* spp.). The recorded sergeants, most probably *A*. *vaigiensis*, typically occurred in loosely congregated shoals feeding on by-drifting plankton in the mid-water. The predatory trevallies were observed patrolling in shoals or small groups.

**Fig 4:**
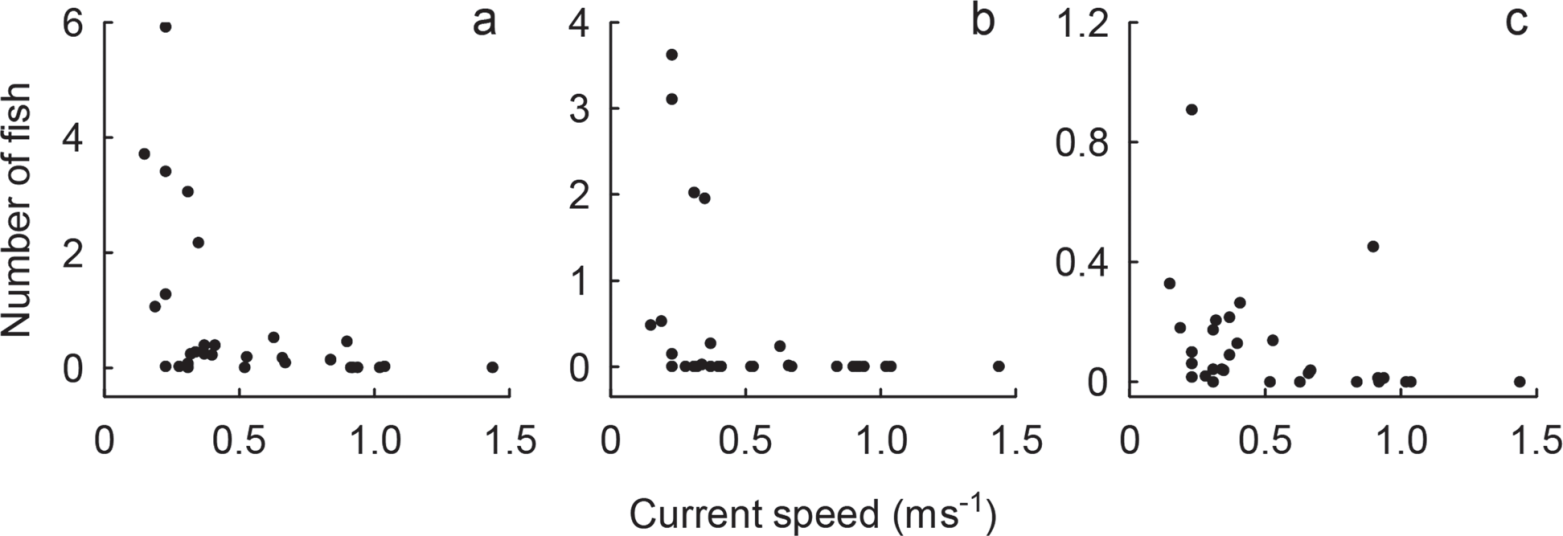
Activity of (a) pelagic associated fish, (b) sergeant fish and (c) trevallies over current speed. Number of fish represents the count of fish entering the recorded video frame during each sample (specimens per m^2^ and 45 min). The negative correlative relationship between fish activity and current speed indicates that pelagic fish at the studied location avoid strong currents.

As illustrated by **Fig. 4**, fish activity varied highly in slow currents and decreased with current speed. A negative correlative relationship (Linear regression: *R* ^2^ = 0.300, *F*_1,28_ = 11.960, *P* < 0.01) between current speed and activity of pelagic associated fish was established. Negative correlative relationships were also found for sergeants (Spearman rank correlation: *ρ* = -0.564, *P* < 0.05) and trevallies (Spearman rank correlation: *ρ* = -0.543, *P* < 0.05) considered alone. These results indicate that the studied fish utilize the current swept habitat but leave or seek shelter when current speed becomes too high for the benefits to outweigh the energy costs of swimming.

**Fig 5:**
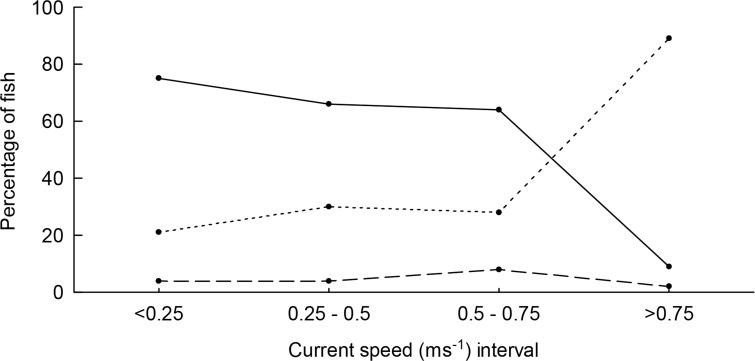
Observed swimming direction for pelagic associated fish over categories of current speed. Solid line indicates counter-current swimming; dotted line indicates along-current swimming; broken line denotes transverse swimming. The results indicate that in strong currents most fish swim along with the flow.

In slow currents most of the fish swam in counter-current direction but the proportion of along-current swimmers increased with current speed (**Fig. 5**). The influence of current speed on swimming behavior was investigated for the two most common fish using logistic regression. It was shown that current speed is a significant predictor of swimming direction (counter-current vs. along-current) for both sergeants (*Abudefduf* spp.) (Logit: N = 60, d.f. = 1, *X* ^*2*^ = 9.327, *ROC* = 0.843, *P* < 0.001) and Brassy trevally (*Caranx papuensis*) (Logit: N = 98, d.f. = 1, *X* ^*2*^ = 49.830, *ROC* = 0.876, *P* = 0.001). This means that the probability of along-current swimming increases with current speed. The breaking points where along-current swimming becomes more probable than counter-current swimming were predicted from the logistic models, with sergeants experiencing a breaking point at 0.65 ms^−1^ and the Brassy trevally having a breaking point at 0.77 ms^−1^. The higher tolerance to current speed among trevallies was expected, due to their larger size and more fusiform body shape. Regardless of species and current speed, very few specimens (<1%) moved tail-first backwards.

It was further shown that current speed predicts swimming depth (bottom vs. pelagic) to some extent for both sergeants (Logit: N = 87, d.f. = 1, *X* ^*2*^ = 9.616, *ROC* = 0.714, *P* = 0.003) and Brassy trevally (Logit: N = 142, d.f. = 1, *X* ^*2*^ = 60.872, *ROC* = 0.837, *P* < 0.001). The probability of fish swimming in the pelagic increased with current speed. The predicted breaking points, where swimming in the pelagic becomes more probable than swimming close to bottom (<2 m above bottom), were 0.65 ms^−1^ for the sergeants and 0.72 ms^−1^ for the Brassy trevally.

### Implications of the field study for fish-turbine co-occurrence

The field study generated three important findings regarding the generic probability of co-occurrence between fish and tidal turbines. Firstly, previous studies indicating a negative relationship between fish presence and current speed were confirmed. Despite high fish activities during slack tide, fish were very rare in currents as strong as 1 ms^−1^. Thus, many species of fish will have very low probabilities of co-occurring with operating tidal turbines, where current speeds typically are 2–3 ms^−1^, simply because they avoid the most forceful currents. Secondly, the findings indicate that fish in strong tidal currents most likely will swim in along-current direction, increasing the probability of entering turbines in the direction of the flow (see **Fig. 3**, high Z-axis probability). Thirdly, the studied fish were increasingly likely to swim at mid water depth as current speed increased. This increases the probability of fish entering turbines at rotor depth (see **Fig. 3**, high Y-axis probability), given that they are present despite the strong current.

In summary, fish of the studied taxa are unlikely to encounter operating tidal turbines even if turbines are installed in their preferred habitat. However, for the few specimens nevertheless swimming in the strong flows, the probability of entering the rotor increases with current speed. The flow velocity where swimming behavior becomes strongly affected was found to be around 0.7–0.8 ms^−1^, which is consistent with previous findings for Pollock [10] and observations of other, unspecified, fish [11]. Literature further indicates that some migrating species, such as Atlantic herring, may be abundant in flows up to about 2 ms^−1^ [8]. These generic patterns can be used to assign approximate probabilities of co-occurrence were case-specific field sampling has not been conducted.

## Avoidance Failure

**Fig 6:**
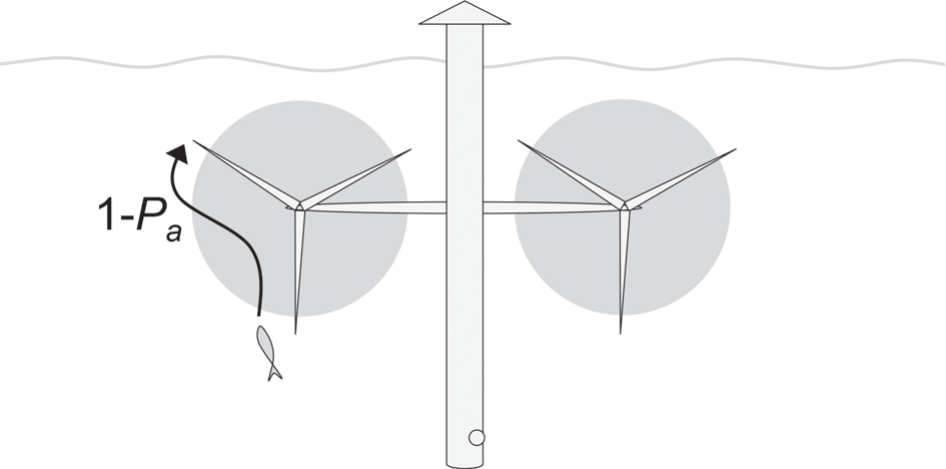
Conceptual representation of avoidance failure (*P*_*a*_). *P*_*a*_ is the probability that a specimen who approaches an operating turbine will fail to avoid the rotor by swimming away.

The probability of avoidance failure (*P*_*a*_) denotes the ability of specimens to detect a turbine and actively avoid entering the rotor swept disc (**Fig. 6**). Avoidance has often been left out of collision risk modeling due to lack of observations from large diameter turbines, although it is generally considered important for the outcome. In this section, we develop models for estimating fish avoidance ability based on the sparse available information on fish behavior in relation to approaching objects. The result is used for obtaining rough estimates of avoidance failure probability.

### Remote detection of turbines

The main remote information sensor systems among fish are vision, hearing, the lateral line system and olfaction. Olfaction is of little use for detecting turbines because turbines are not associated with fish-recognizable chemical compounds (and plumes would not travel counter-current to warn approaching fish). The lateral line system, allowing fish to sense pressure changes in water such as low frequency vibrations [38,39], may assist fish to detect vibrations of an operating turbine at some distance. But for most species, a tidal turbine will first be detected by hearing [21,22]. Fish hearing is generally well developed and allows fish to detect sound over a wide frequency spectrum, with high sensitivity at low frequencies [40]. Underwater sound can be described as sound exposure level (SEL), given as root mean square (RMS) decibels relative a pressure of 1 μPa. The noise emissions from tidal turbines are not yet well established but low frequency (<1 kHz) SEL source levels of 160–175 dB_RMS_ re 1 μPa at 1 m seem expectable [41–43]. Considering sound propagation in water [44], hearing-sensitive fish such as herring may detect an operating tidal turbine at a distance of hundreds of meters. At 10–100 m distance the turbine noise can be expected to induce distinct avoidance among hearing-sensitive fish. For most fish, however, turbine noise will not be unbearably loud even at close distance [22,42,45]. Remote detection of unrecognized noise affects different fish differently [46–49]. While avoidance cannot be expected unless the noise is unbearable, increased alertness may take place. Regarding turbine noise in particular, fish of different species have been observed shoaling close to tidal turbines in operation [8,10]. For sharks, which are generally attracted to low frequency sound (10–800 Hz) [50], the noise of a tidal turbine may even attract specimens within hearing range. In conclusion, hearing-sensitive fish may be repelled at distance, but most species will not initiate avoidance reactions as a cause of turbine noise [22].

Visual sensing is highly important among fish and most species have particularly well developed ability to differentiate contrasting objects from the background [51]. Large moving object are known to evoke visually mediated startle responses [52] and it can be assumed that the visual stimuli of a turbine in motion will be an effective trigger of fish avoidance, as has been clearly shown for fish approached by fishing gear [49,53]. Turbidity and light conditions affect the visual sensing detection range. Fish can adjust the sensitivity of the eye with changing light conditions and it is difficult to predict reductions in detection range solely based on available light [51]. Fish tank experiments where predator-prey detection range was compared over daylight (20 lx) and lowlight (0.1 lx) conditions indicated reductions of about 70–80% (with high variations among species and individuals) [54]. However, evidence suggests that in very dark conditions, fish will only react to an approaching object once in physical contact or detected by the lateral system at very close range [53].

### Different ways of fish avoidance

The few available studies on fish behavior around small tidal turbines have demonstrated some different responses. Viehman [8] monitored fish movements around a horizontally positioned cylindrical turbine in currents of 1–2 ms^−1^ and demonstrated that approaching fish either entered the turbine or avoided the turbine by altering the path of swimming. Avoidance actions were categorized as (1) turning in reverse direction and swimming away counter-current, or (2) turning into a divergent direction and swimming past the turbine. During night, fish–turbine interactions were more common and avoidance actions took place closer to the turbine, indicating that avoidance was initiated by visual stimuli. Hammar *et al*. [9] recorded fish movements around a similar but vertically positioned turbine. That study was restricted to daylight conditions and currents below 1.5 ms^−1^. It was shown that fish generally avoided the turbine and kept a greater distance from the turbine as current speed increased. Fish startled by the turbine were observed to undertake quick bursts in divergent directions. As the observations from these two studies are associated with small turbines (Ø ≈ 1 m), they may only partly be extrapolated to large turbines (Ø = 5–20 m). The only observation of fish–turbine interactions at a larger tidal turbine concerns the Verdant Power turbine (Ø = 5 m) [11], where a shoal of small fish approached the rotor and diverged just before encountering.

As suggested by Wilson *et al*. [13], the behavior of fish approached by fishing trawls, which move at 1.5–2 ms^−1^, can be an informative analogy to fish behavior at large tidal turbines. Once the trawl otter board is visually detected, fish turn around and swim away at the same pace as the moving hazard, without sprinting to gain distance from the moving hazard [49]. Wardle [49] showed that by taking a reverse direction where the otter board is still kept in the rear view (~25° diversion from straight reverse direction), the fish can keep an eye on the hazard and slowly move in the transverse direction until ending up on the other side of the otter board, that is, either outside or inside the mouth of the trawl. Fish trapped in front of the trawl mouth swim along with the trawl until exhausted, instead of overtaking it (even if physiologically possible) [49]. This restricted avoidance behavior is thought to be a way of avoiding unnecessary energy losses. Similar sustained swimming has been observed among fish trapped in front of hydropower turbine intakes [55] and in laboratory conditions [56]. Once exhausted, the fish turn and swim towards the approaching hazard.

Based on these observations, there seems to be two basic avoidance strategies for fish approaching and detecting a tidal turbine: (1) the ‘reverse’ strategy and (2) the ‘diverge’ strategy. Taking the ‘reverse’ strategy, the fish will turn around and swim in the reverse direction against the current, but askew by a small angle, at about the same speed as the current until the skewed course has moved the fish to safety or until the fish exhausts and falls back into the rotor swept disc. Taking the ‘diverge’ strategy, the fish will diverge and swim fast towards the outer edge of the rotor until reaching safety, or being pushed through the rotor by the current.

**Fig 7:**
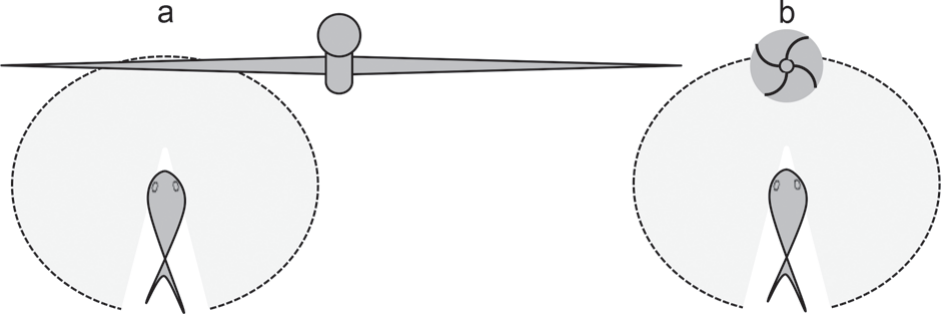
Fish visual coverage of (a) a large diameter turbine, and (b) a small object. In limited visibility a fish may be able identify a small object, like a vertical-axis turbine or a trawl otter board, at approximately the same time as it comes in view. But a very large object, like a horizontal-axis turbine, may never be fully within view and approaching fish cannot be expected to know the shortest path of avoidance. Shaded semi-circles indicate fish fields of view.

An important difference between turbines and fishing gear is that the turbine may be too large for a fish to see the whole of it, given limited visibility and that many fish are short-sighted (**Fig. 7**). Therefore, it cannot be assumed that fish will always choose the shortest way to safety.

### Models describing fish avoidance

Although the two outlined avoidance strategies are simplified stereotypes of fish behavior, they may, in the absence of quantitative data on fish behavior around large tidal turbines, be useful for indicative modeling of the probability of avoidance failure (*P*_*a*_).

**Fig 8:**
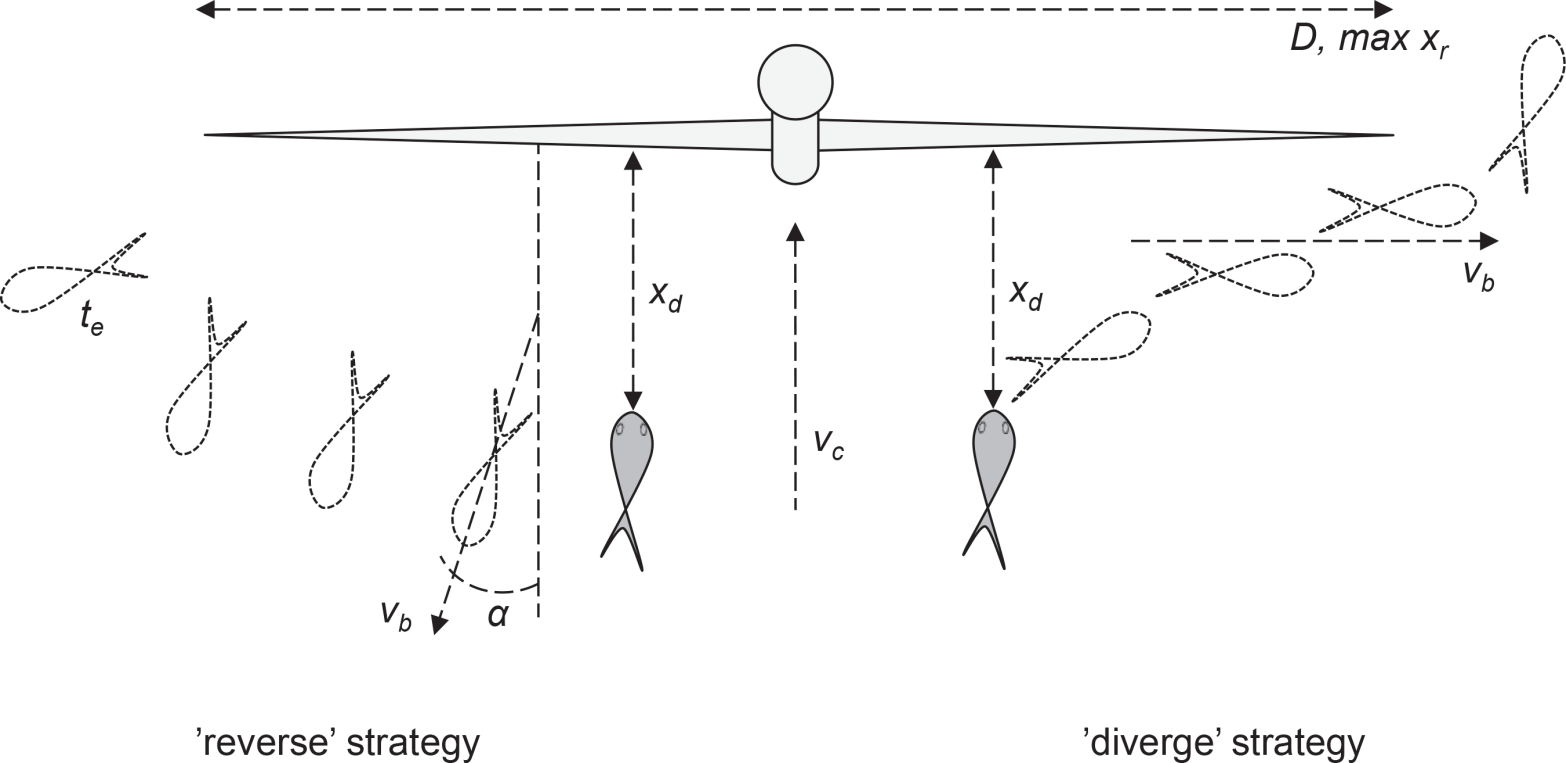
Representation of the two modeled avoidance strategies for a fish approaching a turbine. In these simplified models of fish avoidance *D* denotes the rotor diameter, defining the maximum swimming path of avoiding fish. *x*_*r*_ is the random position on the rotor disc where the fish appears when approaching the turbine and *x*_*d*_ is the distance at which the fish detects the turbine. *x*_*r*_ is the required swimming path, defining the distance that the fish has to swim to reach safety. *v*_*b*_ denotes the fish burst speed and *α* is the angle that reversing fish take in order to keep the turbine in the rear view while swimming away. *t*_*e*_ is the time to exhaustion for reversing fish, which defines the time in which the fish has to cover the required swimming path to safety (outside the rotor swept disc).

The ‘reverse’ avoidance strategy implies that the fish turns and swims in the reverse direction (*α*) away from the turbine after having detected it at the maximum detection distance (*x*_*d*_), related to its visibility in the water (**Fig. 8**). If having the physiological capability, it will keep swimming at a speed that just compensates for the speed of the current (*v*_*c*_) and will fail the avoidance attempt if the time taken to cover the required swimming path (*x*_*r*_) in transverse direction of the current, given that the reverse direction *α* is longer than the time to exhaustion (*t*_*e*_). The required swimming path (*x*_*r*_) is the distance to safety from the random position on the rotor where the fish appears when detecting the turbine. With a reverse direction of 25°, the time taken to move from one blade tip to the opposite of a 20 m diameter turbine (the extreme case of fish “choosing the wrong avoidance direction”) is around 20 seconds for fish swimming against a current of 2 ms^−1^, and less at higher speeds. Fish with the capacity to swim faster than the current for more than about 20 seconds will therefore typically succeed to avoid a turbine if taking the ‘reverse’ avoidance strategy. But a fish that cannot keep up against the current will have to swim at burst (maximum) speed (*v*_*b*_) until it covers the required swimming path or fail the avoidance attempt (by getting exhausted or being pushed through the turbine before the required path is covered). If the time it takes to cover the required swimming path while burst swimming in the skewed reverse direction is longer than the time it takes to be pushed backwards into the turbine, or getting exhausted, the avoidance attempt will fail (**Eq. 4**). The detection distance defines the initial distance between the fish and the turbine.

$$\min\left(t_{e},\frac{x_{d}}{v_{c}-v_{b}\;\cos\;\alpha }\right)<\frac{x_{r}}{v_{b}\;\sin\;\alpha }$$ (Eq. 4)

The alternative ‘diverge’ avoidance strategy implies that a fish burst swims towards the edge of the turbine once it is detected (**Fig. 8**). This way of avoidance fails if the time it takes to cover the required swimming path is longer than the time it takes for the current to push the fish into the turbine (**Eq. 5**). Here, it is assumed that the fish rationally invest all swimming effort in the direction transverse to the current (towards the edge of the rotor swept disc).

$$\frac{x_{d}}{v_{c}}<\frac{x_{r}}{v_{b}}$$ (Eq. 5)

The two avoidance strategies—‘reverse’ and ‘diverge’—are here described with considerable simplicity, not accounting for the time it takes for the fish to react, turn and accelerate. However, the startle response in escaping fish, characterized by a short acceleration to maximum speed, is fast and typically lasts only for about one second [57]. The models further assume that the fish only moves in the horizontal plane and attempts to avoid the whole rotor swept disc, not considering slipping through were the rotor blades moves slow close to the nacelle. Hereby, the models can be considered conservative.

In order to estimate avoidance failure (*P*_*a*_) by orders of magnitude, we implemented Monte Carlo simulations of the two proposed models for the two common fish taxa observed in the previously described field study: Brassy trevally (*C*. *papuensis*) and sergeant fish (*Abudefduf* spp.). We ran the simulations for turbines with rotor diameters of 5 m [58] and 20 m [59], for current speeds of 2 and 3 ms^−1^ and for two different visibility conditions (daytime and lowlight). Uncertain and naturally varying model parameters were assigned as probabilistic distributions and binomial model outputs (success/failure for 10^5^ model runs) were averaged into probability of avoidance failure. Applied assumptions and parameter distributions are provided as supporting information (**S1 Table**). Sensitivity analyses were conducted by changing each parameter by ±50% [60].

### Modelling results for avoidance failure

The model implementation of the two avoidance strategies indicated that the probability of avoidance failure varies over about two orders of magnitude for the tested fish taxa, avoidance strategies, current speeds, light conditions and turbine diameters (**Table 2**). In most tested cases, *P*_*a*_ is above 0.1 and in lowlight conditions *P*_*a*_ is above 0.75, apart from trevallies taking the ‘reverse’ strategy. Failure rates increase with larger turbine diameters, faster currents and at lowlight conditions. For all tested scenarios, the larger and faster trevallies are more successful than the smaller sergeant fish. Similar results were found for fish at the small vertical-axis turbine investigated by Viehman [8]. For trevallies, the ‘reverse’ strategy was constantly the more effective way of avoidance whilst for the sergeants the two strategies were more equal. Which of the tested avoidance strategies that is more likely among different species is not known. In the study by Viehman [8], both strategies were observed by the same species.

**Table 2:** Computed avoidance failure (Pa) for the two modelled avoidance strategies and the two investigated fish taxa.

**Fish and avoidance strategy**	***D* = 5 m**	***D* = 5 m**	***D* = 20 m**	***D* = 20 m**
	***v*_*c*_ = 2 ms^−1^**	***v*_*c*_ = 3 ms^−1^**	***v*_*c*_ = 2 ms^−1^**	***v*_*c*_ = 3 ms^−1^**
***Daylight***				
**Trevally ‘reverse’**	0.02	0.11	0.13	0.39
**Trevally ‘diverge’**	0.04	0.12	0.49	0.63
**Sergeant ‘reverse’**	0.28	0.58	0.73	0.88
**Sergeant ‘diverge’**	0.24	0.42	0.74	0.83
**Lowlight (10% of daylight)**				
**Trevally ‘reverse’**	0.21	0.56	0.30	0.68
**Trevally ‘diverge’**	0.78	0.85	0.94	0.96
**Sergeant ‘reverse’**	0.90	0.96	0.97	0.99
**Sergeant ‘diverge’**	0.91	0.94	0.98	0.98

Probabilities of avoidance failure (*P*_*a*_) were modelled for different settings of rotor radius (*D*) and current speed (*v*_*c*_). The results can be interpreted as the mean probabilities of avoidance failure for a fish randomly drawn from the populations described by the probability distributions for biological parameters, based on literature and field study data (see **S1 Table**).

The results show that the most effective ways of reducing avoidance failure rates would be to reduce the rotor diameter, lower the current velocity, increase the swimming capacity or inform larger fish to choose the ‘reverse’ strategy (**Table 3**). Among these suggestions, only promoting smaller rotors could be a possible option. Increasing the detection distance may not be as effective *per se*, but it is a much more technically feasible option. However, as can be seen in **Table 3**, and by comparing the failure rates between daylight and lowlight conditions in **Table 2**, detection distance needs to be greatly increased to have significant influence on avoidance failure in lowlight conditions. Hence, luminous or illuminated rotors may be required.

**Table 3:** Model sensitivity analyses.

**Parameter**	***D, x_r_***	***v_c_***	***x_d_***	***v_b_, L_f_***	***α***	***t_e_***	***D, x_r_***	***v_c_***	***x_d_***	***v_b_, L_f_***
***Daylight***										
**+50%**	+22	+78	−22	−69	−2	±0	+18	+18	−22	−22
**−50%**	−37	−89	+33	+113	+21	+7	−40	−40	+28	+28
***Lowlight***										
**+50%**	+3	+35	−4	−56	+11	±0	+1	+2	−2	−2
**−50%**	−8	−85	+5	+43	−5	±0	−4	−4	+2	+2

Proportional (%) change of avoidance failure (*P*_*a*_) for changes in parameters (±50%). The sensitivity analyses were based on Brassy trevally performances at a 20 m rotor in 3 ms^−1^ current. The model insensitivity to changes in *t*_*e*_ is explained by the generally low detection distance in comparison to fish endurance (few fish become exhausted before reaching safety or entering the rotor).

In conclusion, the developed models suggest that avoidance failure should be assumed high for any fish with lower swimming capacities than the current speed. Some large predatory pelagic fish can swim extraordinarily fast [61,62] and would manage to avoid turbines under any current speed and turbine diameter. But if the turbine is not detected in time for a sharp turn and acceleration, which may take a few seconds for large fish [63], avoidance will nevertheless fail.

## Hazard Zone

**Fig 9:**
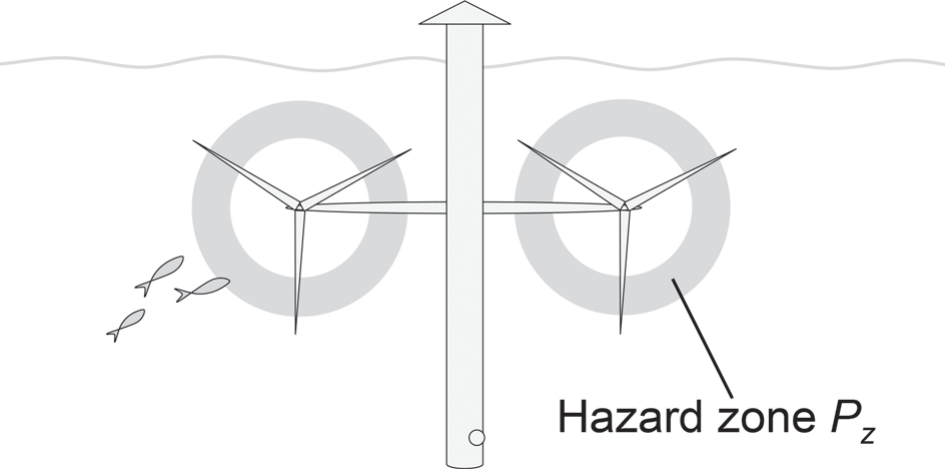
Conceptual representation of the hazard zone (*P*_*z*_). *P*_*z*_ is the hazardous part of the rotor as a proportion of the full rotor swept disc.

Only part of the rotor swept disc constitutes a hazard zone (*P*_*z*_) where animals may suffer severe injury by the turbine (**Fig. 9**). Even for fast rotating turbines, the inner parts of the rotor blades move too slowly to cause damaging collisions or hydrodynamic stress to most animals. According to Cada *et al*. [64], this inner safety zone typically extends from the nacelle to the radius where blades move at 5 ms^−1^. For turbines with blade tip velocities of 10 ms^−1^, a typical upper limit for large tidal turbines [65], this inner safety zone corresponds to 25% of the rotor swept disc.

Additionally, the obstruction of flow caused by a turbine generates a hydrodynamic bow in front of the rotor. This means that incoming water will be forced slightly towards the edges of the rotor and specimens approaching the outer parts of the rotor swept disc will consequently be pushed away [22]. The extent of this outer safety zone varies among turbine designs. For a small turbine (Ø = 1 m), Pearson *et al*. [22] reported an outer safety zone corresponding to 20% of the rotor swept cross-sectional disc while Cada *et al*. [64] reported estimates of about 10% for larger turbines (Ø = 5 m). Based on this information, the hazard zone of a large tidal turbine can be approximated to about two-thirds of the rotor swept disc. A more detailed analysis of relationships between blade velocity, blade thickness and hazard for fish has been carried out by Romero-Gomez and Richmond [17].

## Blade Incident

A specimen passing through the rotor swept disc may collide with a rotor blade if any of the rotor blades cut through the swimming trajectory at the same time as the specimen passes through. This probability, here called blade incident (*P*_*b*_), has been modeled in several previous studies on collision risks at hydrokinetic turbines [11,13,14,17] and conventional hydropower turbines [18–20]. Using a commonly applied model variant, proposed by Schweizer *et al*. [14], *P*_*b*_ can be calculated from the number of turbine blades *n*, the rotational speed *R* (rpm), the angle of attack *α*_*a*_ formed by the fish in the water flow and the axial direction of the turbine, the fish total length *L*_*f*_ (m), and the combined speed of current and fish *v*_*cf*_ (ms^−1^) (**Eq. 6**).

$$P_{b}=\frac{n\times \left(\frac{R}{60}\right)\times \cos\left(\alpha_{a}\right)\times L_{f}}{v_{cf}}$$ (Eq. 6)

*P*_*b*_ increases with fish length and decreases with current speed [14]. The rotational speed of the turbine is particularly influential. It would be almost impossible for large animals, such as barracudas and sharks, to pass through turbines with high rotational speed without colliding with rotor blades [15].

Both of the two biological model parameters—swimming speed and body orientation—will be affected by previously undertaken avoidance behavior. A fish undertaking the ‘reverse’ avoidance strategy prior to entering the turbine will, unless already exhausted, be swimming counter-current, thus both reducing its speed through the turbine and maximizing its relative body length, which will increase *P*_*b*_. But fish undertaking the ‘diverge’ avoidance strategy swim parallel to the turbine blades, thus minimizing their relative body length and keeping approximately the same speed as the current. This behavior strongly reduces the time taken for the fish body to pass through the rotor, thus generating a comparatively low *P*_*b*_. For example, implementing **Eq. 6** on Brassy trevally at a two-bladed turbine with 14 rpm rotational speed in 2 ms^-1^ current generates *P*_*b*_ = 0.52 if it has failed the ‘reverse’ avoidance strategy and is swimming against the current, while *P*_*b*_ = 0.02 if it has attempted the ‘diverge’ avoidance strategy and swims in parallel to the rotor blades.

## Evasion Failure

Specimens that pass through a rotor and are about to collide with a blade may still prevent collision by undertaking a final close-range evasive maneuver around the blade [11,13,14]. This type of action has been confirmed by video footage of small reef fish at a vertical-axis turbine rotor with 2 ms^−1^ blade-tip speed [9]. Evasive maneuvers, characterized as a startle response triggered by vision or the pressure sensitive lateral line system, are likely to be most effective among small agile fish with quick acceleration per body length. Elaborating on experiences from conventional hydropower turbines, evasion of small fish may be further aided by the drag force exerted on the fish body by the water that is forced around the side of the rotor blade. Hence, small fish can be drawn around a rotor blade without colliding, as noted by Pearson *et al*. [22]. Large fish have comparatively low agility and their bodies are less affected by hydrodynamic drag forces around the blades. Evasion failure (*P*_*e*_) can therefore be expected to be high for large fish.

## Blade Damage

Direct collision with rotor blades can cause different kinds of injury, some of which are more likely to cause immediate death and others potentially causing demise with time. Samples from tidal barrages with low head hydraulic turbines have shown signs of maceration, laceration, abrasion and contusion on marine fish [66]. The level of injury depends on the blade, the animal and circumstantial conditions [22]. The body orientation during impact has also a large influence on the level of injury. Among fish known to have collided with conventional hydropower rotor blades, up to 50% of the specimens of some species have turned out unharmed because only flexible body parts have been hit [18]. In contrast to conventional low head turbines, many tidal turbine devices have a blunt leading edge, which will additionally prevent laceration injuries. At strike, fish with low mass and inertia sustain less physical injury than large heavy animals.

Survival tests on small fish in hydrokinetic turbines have all shown very low mortalities [67,68]. Due to experimental designs, the reasons for low mortality have not been well distinguished among avoidance, close-range evasion, blade damage and hydrodynamic stress tolerances. The largest turbine tested was a Ø = 4 m horizontal-axis turbine [67]. Five different species of freshwater fish, with body lengths below 0.7 m, were entrained and survival rates were >99%. However, the blade-tip speed was low (<5 ms^−1^) and the results may thus not be applicable for larger turbines.

Very little is known about potential injuries on large animals colliding with rotors. Analogous events may be collisions between marine animals and the keels and bows of marine vessels [13]. However, research on this topic is scarce and little information is available regarding fish. Concerning marine mammals and chelonians, it is known that while all collisions are not critical, fatalities do occur [69,70].

In conclusion, the probability of fatal blade damage (*P*_*d*_) differs among turbines, species and sizes, with substantially higher blade damage among large specimens.

## Hydraulic Stress

In addition to mortality caused by physical collision, hydraulic forces around the blades may cause serious injury. Examples from low head turbines include pressure drops behind the blades, shear stress, where adjacent water streams move at different velocities and cavitation at fast-moving rotor blades. These hydraulic forces can cause both internal injuries and tissue ruptures such as torn off body parts [66]. In a field experiment at a small conventional hydropower turbine, fatal injuries caused by hydraulic stress were shown among 8% of the passing fish [71]. Most hydrokinetic turbines have more open flow designs (limited pressure drop) and much lower rotational speeds (no cavitation) compared to hydropower turbines. Therefore, exerted hydraulic forces are likely to be less damaging and the probability of hydraulic stress (*P*_*s*_) can be expected to be low even for small fish. This is further supported by the evidence that small fish entrained through small but fast revolving hydrokinetic turbines have high survival rate [68].

## Example of Collision Risk Modelling on Field Study Species

For demonstrative purposes, the fault tree based collision risk model was tested for Brassy trevally investigated in the field study, using the above discussed literature and findings. Since the population size of Brassy trevally at the field study location is unknown, an arbitrary local population size of 10 000 specimens was assigned. Given this assumption, the calculated collision risk is strictly hypothetical. Calculations were based on the hypothetical installation of 1 turbine with a 20 m diameter rotor (swept rotor disc: 314 m^2^) and a constant rotational speed of 14 rpm above the cut-in speed of 0.75 ms^-1^. Tidal current distributions were based on a semidiurnal tide location with 1.2 ms^-1^ average speed and 3.5 ms^-1^ maximum spring speed (MSS) [72]. Parameter probabilities and assumptions are given in **Table 4**, with the *yearly N*_*TM*_ based on six settings of representative tidal and light conditions.

**Table 4:** Implementation of the fault tree based collision risk model presented in this paper.

**Model component**	**Day**	**Day**	**Day**	**Night**	**Night**	**Night**	**Source and assumptions**	**Involved parameters**
	**<0.75 ms^-1^**	**0.75–1.5 ms^-1^**	**>1.5 ms^-1^**	**<0.75 ms^-1^**	**0.75–1.5 ms^-1^**	**>1.5 ms^-1^**		
***P*_*o*_*×P*_*p*_ Array passage and co-occurrence**	1.77×10^–3^	2.22×10^–3^	4.19×10^–5^	1.77×10^–3^	2.22×10^–3^	4.19×10^–5^	Field study results* for *C*. *papuensis*, not accounting for differences in fish activity between day and night	Fish activity (N_fish_ m^-2^ h^-1^), rotor swept area (m^2^), population size (N_fish_), assessment unit (h)
***P*_*a*_ Avoidance failure**	0.02	0.28	0.49	0.72	0.90	0.94	Model for ‘diverge’ avoidance (Eq. 4) applied on *C*. *papuensis*	Detection distance (m), current speed (ms^-1^), rotor radius (m), fish burst speed (ms^-1^)
***P*_*z*_ Hazard zone**	0	0.65	0.65	0	0.65	0.65	Inner (25%) and outer (10%) safety zones at currents above turbine cut-in speed (0.75 ms^-1^)	Proportional (%), unknown parameters
***P*_*b*_ Blade incident**	0	0.03	0.02	0	0.03	0.02	Blade incident model (Eq. 6) applied on *C*. *papuensis* while undertaking ‘diverge’ avoidance, above turbine cut-in speed	Number of blades (n), Rotational speed (ns^-1^), angle of attack (°), fish length (m), current speed (ms^-1^)
***P*_*e*_ Evasion failure**	0	0.50	0.50	0	0.75	0.75	Assuming 50% and 75% evasion failure at day and night respectively, for *C*. *papuensis* above turbine cut-in speed	Proportional (%), parameters not established
***P*_*d*_ Damage**	0	0.75	0.75	0	0.75	0.75	Assuming no fatal injury to the flexible tail end of fish (damage only at currents above turbine cut-in speed)	Proportional (%), parameters not established
***P*_*s*_ Hydraulic stress**	0	0	0	0	0	0	Assuming that *C*. *papuensis* is too large and sturdy for hydraulic stress to be severe	Proportional (%), parameters not established
***P*_*TM*_ Turbine mortality**	0	3.80×10^–6^	7.50×10^–8^	0	1.83×10^–5^	2.16×10^–7^	Calculation of hour-based *P*_*TM*_ (Eq. 2)	(*P*_*p*_), *P*_*o*_, *P*_*a*_, *P*_*z*_, *P*_*b*_, *P*_*e*_, *P*_*d*_, *P*_*s*_
***Hourly N*_TM_**	0	0.038	0.001	0	0.183	0.002	Multiplying the hour-based *P*_*TM*_ with population size	*P*_*TM*_, population size (N_fish_)
**Hours**	1460	1460	1460	1460	1460	1460	Light and tidal current settings at a location with a semidiurnal tide with 3.5 ms^-1^ MSS	Hours per year (h)
***Yearly N*_TM_**	0	111	2	0	534	6	Multiplying the *Hourly N*_*TM*_ with the number of hours per year for each setting (result: *yearly N*_*TM*_ = 654 specimens)	Hourly turbine mortality (hourly N_TM_), hours per year (h)

In this example the yearly loss of fish is approximately 650 specimens. The example is based on sampled biological data for Brassy trevally (*Caranx papuensis*) and literature-based assumptions. For explanations of model components and details on parameter assumptions, see each respective section in the main text.

*The number of *C*. *papuensis* moving in along-current direction in the pelagic per hour was multiplied by the rotor swept area and divided by population size to determine the hourly probability of each specimen from the population to come across the rotor. In-data for fish activity (N_fish_ m^-2^ h^-1^) at increasing current intervals: 0.14; 0.06; 0.001. In-data for along-current swimming (%): 31; 90; 100.

The result from the model implementation indicates a yearly loss of about 650 specimens. This number has to be related to the population size and dynamics in order to assess the risk for the population (which is not meaningful here due to the arbitrarily set population size). Importantly, the computed example indicates that the number of fish mortalities is highest at medium current speeds and that fish behavior (such as swimming activity in the pelagic at different current speeds, avoidance and evasion) plays an important role for the probability of turbine mortality. Among the Brassy trevallies that do approach the turbine while it is operating the average survival rate is approximately 99.5%.

## Discussion

### General findings regarding collision risks for fish

The general collision risk model presented in this paper can be applied for a number of different turbine designs, species and environmental conditions. As research progresses, the model and equations supporting each model component can be refined and more case-adapted to specific species, sites and turbine designs. The discussion on collision risks for fish and large tidal turbines presented in the previous sections shows that there are uncertainties associated with each model component. Some indicative and potentially important findings can nevertheless be extracted.

The first model component, array passage, is strictly site-specific and can hardly be discussed in general terms, apart from stating the obvious fact that an array passage above zero is a prerequisite for collision. But the other model components indicate contrasting results for different size groups of fish. Large fish seem to have high probabilities of collision (*P*_*c*_) compared to small fish, including high probabilities of blade incident (*P*_*b*_), evasion failure (*P*_*e*_) and blade damage (*P*_*d*_). Since hydraulic stress (*P*_*s*_) is expected to be low at open-flow tidal turbines in general, it can be expected that the probability of turbine injury (*P*_*i*_) as a whole is low for small fish but substantial for large-sized species.

The probability of turbine entry (*P*_*t*_), however, is rather species-specific. The findings regarding fish utilization of strong flows indicate that co-occurrence (*P*_*o*_) between operating turbines and many fish species is very low. This and other studies [8,10,11] support that currents above 2 ms^−1^ are not frequently utilized by fish of any investigated species. However, for fish nevertheless encountering operating turbines avoidance, failure (*P*_*a*_) can be expected to be high unless the fish can swim faster than the current speed. Large fast-swimming fish will be able to avoid the hazard if the rotor is detected in time for reaction and acceleration. But since many fish do not respond to moving objects until they are within visual range [49,53], turbines may be problematic for large fish at lowlight conditions.

The findings consequently indicate that small individuals are unlikely to come to harm from tidal turbines while large fish seem to be at considerable risk where they enter the strong flows at a turbine location during night. Size is a continuous variable and there is no distinct boundary between small and large fish. For clarity, large fish can in this context be thought of as large predatory fish, such as barracuda, shark, billfish and tuna, and large planktivorous fish such as sunfish, whale shark and basking shark. This latter category might be at highest concern because they are slow swimmers, reducing avoidance abilities.

It should be noted that turbine design has a large influence on the potential mortality. Small turbines are easier to avoid than large ones, and slow rotational speeds reduce the probability of all model components associated with turbine injury. Only turbines with rotors moving fast and across a large volume of water (i.e. having a large diameter) may therefore pose risks as outlined above. Among the many developing turbine designs, the Deep Green design [73] is distinguished by the fact that it moves very fast (>10 ms^-1^) and has a very large diameter (the tether being about 100 m long). This design also operates in relatively slow currents (1 ms^−1^) where fish activity is higher than in stronger currents. The installation of such turbines in areas frequented by large fish of vulnerable populations should therefore be carefully assessed with regards to ecological risks before installation. These findings support previous studies where apex predators have been identified as the most vulnerable to tidal turbine collision risks [5,74].

As illustrated by the model implementation example, it is important to have a thorough understanding of fish movements in the pelagic prior to any thorough turbine collision risk assessment. The example generated a survival rate of 99.5% for Brassy trevallies actually approaching an operating turbine (thus not accounting for natural swimming patterns; *P*_*p*_ and *P*_*o*_). The passive models by Romero-Gomez and Richmond [17] generated corresponding survival rates between 87% and 99% for similarly sized fish at current speeds of 1–3 ms^-1^. The results are thus in the same order of magnitude and differences can probably be attributed to active avoidance. However, not to include fish natural swimming patterns in relation to current speed may strongly overestimate collision risks as shown in this study.

### Limitations

Currently, there is no available data to be used for model validation and, as described in previous sections, the many uncertainties mean that results must be understood as indicative. More research and monitoring on animal–turbine interactions are needed. Once data are available validation can take place, both for separate model components and for the complete model. Such future validations should cover several different taxa.

Only collision risks for fish have been examined in this paper. Fish is, however, a very large and variable organism group, in terms of more than size and swimming ability. For instance, while many fish are short-sighted, some fish have highly developed vision. For this latter category, the estimated visual detection distances used in the avoidance models would be misleading. Moreover, many small fish occur in dense shoals, which may strongly affect their ability to detect and avoid turbines. Viehman [8] reported that whole shoals of small fish were observed to enter the small turbine under study and it has been argued that densely shoaling fish are likely to have lower avoidance ability than solitary fish [13]. To parameterize the factor of shoaling would be an important future development of the avoidance failure and evasion failure model components.

Other animal groups that need to be addressed include marine mammals, chelonians and diving sea birds. Based on the findings for large fish in this paper, it cannot be ruled out that arrays of large tidal turbines would pose risks to vulnerable populations of these animal groups. However, there are many important differences regarding both physical and behavioral traits. For discussion on collision risks from tidal turbines to different marine mammals see for example Wilson *et al*. [13], Copping *et al*. [21], Sparling and Lonergan [16] and Wilson and Carter [75]. Collision risks for birds are discussed by Wilson *et al*. [13] and Langton *et al*. [76]. To the knowledge of the authors, turbine collision risks to chelonians have not yet been addressed although these animals often occur in current-swept areas. The general model-design presented here should however be able to contain further components and refinements allowing for the coverage of other groups of organisms exposed to risks from turbines. In a similar way the general model-design also enables for adapting the model to other hydrokinetic turbines, different from those addressed in this paper, by making adjustments to single model components.

### Technical options for risk reduction

Based on the findings of this synthesis paper, ecological risks related to tidal turbines mostly concern large animals and large-diameter turbines with fast-moving rotors. The collision risk model can further be used for identifying potentially effective risk reducing options. Risk mitigation for large animals might be most effective by reducing the probabilities for blade damage (*P*_*d*_) and blade incident (*P*_*b*_). This can be achieved by rating the turbines for lower rotational speeds.

Another way of risk reduction for large animals is to make sure that animals detect the turbine (and perceive it as a hazard) at sufficient distance, so that avoidance actions can be initiated in time, even in lowlight conditions. Regarding visual detection, rotor coloration should be of stark contrast to the surrounding water. The most distinguishable color differs among environmental conditions and depths [77]. However, given that many pelagic fish have a narrow spectral sensitivity or are effectively color blind, a white coloration against the dark background is likely to be more effective than other colors. Even white color requires available light and, as discussed above, it is possible that luminous or illuminated rotors would be the most effective option. Other means of preventing large animals from entering tidal turbines include acoustic warning systems, which have been suggested for repelling marine mammals at tidal turbines [75], and electric deterrence systems for repelling sharks [78]. For all such active warning systems it is important to carefully evaluate the potential negative effects associated, given the possibly expansive growth of the tidal power industry. For instance, enhanced avoidance may affect migration patterns for both species targeted by the avoidance system and species unintentionally disturbed by such a system. A less intrusive option is to use passive alert systems which can detect the approach of specific animals [79]. Hereby it becomes possible to slow down the turbine or activate repelling warning systems once specimens of protected species come close.

## Supporting Information

S1 Table Assumptions and probability distributions for avoidance failure modelling. (DOCX)
