# Supplementary material for: A Probabilistic Model for Hydrokinetic Turbine Collision Risks: Exploring Impacts on Fish
Source: PLoS One. 2015 Mar 2;10(3):e0117756. doi: 10.1371/journal.pone.0117756 (PMC4346259; doi:10.1371/journal.pone.0117756)
Supplement: S1 Table — (DOCX) [file pone.0117756.s001.docx]

Assumptions and probability distributions for avoidance failure modelling

The suggested models for estimating avoidance failure (*P_a_*) based on the ‘reverse’ and ‘diverge’ avoidance strategies were implemented as Monte Carlo simulations. Data was derived from literature and from the field survey presented in the main text as summarized in **Table S1** and explained below.

The models were implemented for brassy trevally (*C. papuensis*) and sergeant fish of the genus *Abudefduf*, for two different turbines with rotor diameters of 5 m [1] and 20 m [2] and for current speeds (*v_c_*) of 2 and 3 ms^−1^.

Detection distance (*x_d_*) data were derived from visibility estimates from the field survey. Although this video-based visibility measure may not accurately mirror fish vision, the available data were assumed to give a decent approximation for daytime light conditions in a tidal current environment with natural turbidity variation. Lowlight detection distance was approximated to 90% of the daytime estimates. This is meant to represent conditions during a bright night, based on available studies on fish detection distance in varying light conditions [3,4].

The required swimming path (*x_r_*) was given a distribution representing a random positioning within the rotor swept disc, with the possibility of swimming in any horizontal direction. That is, the fish do not know which direction offers the shortest path out. This simplification has support in the notion that fast-starts among escaping fish are typically directed in the horizontal plane [5]. For fish undertaking the ‘reverse’ avoidance strategy the deviation from a straight reverse course (*α*) was assigned a uniform distribution between 20° and 30° based on Wardle [6].

The burst speed (*v_b_*) capacities of the two fish taxa were described as triangular distributions centered on the maximum observed speed (relative to the moving water body) in the field survey and framed by previous findings on fish burst speed capacities, most species having a burst speed capacity of 5–10 body lengths per second (BL s^−1^) [7-9]. In relation to body length, small fish are generally faster than large ones, which was also found in the field survey (brassy trevally: 7 BL s^−1^; sergeant fish: 8 BL s^−1^). Fish swimming at burst speed use white myotomal muscles and soon reach exhaustion. Based on literature on the topic [5,7,8] the time to exhaustion (*t_e_*) was assigned a uniform distribution of 15–20 seconds. Here it should be noted that the swimming endurance is much higher for fish swimming slower, and many fish can swim just below burst speed for several minutes [7]. Finally, fish length (*L_f_*) distributions for the two tested taxa were assembled from the field survey.

**Table S1.** **Assumptions and probability distributions for avoidance failure modelling.**

| **Parameter** | **Assigned distribution** | **Source** |
| --- | --- | --- |
|  |  |  |
| Turbine diameter, *D* (m) | Fixed value, 5 or 20 | Verdant Power HKPS [1], SeaGenS Mk2 [2] |
| Current speed, *v_c_* (ms^−1^) | Fixed value, 2 or 3 | Turbine rated current speeds |
| Daytime detection distance, *x_d_* (m) | Normal (µ = 4.650; σ = 1.229) | Field study |
| Lowlight detection distance | Fixed value, 0.1 *x_d_* | Extrapolation from [3,4] |
| Required swimming path, *x_r_* (m) | Triangular (min = 0; peak = 0; max = D) | Simplification of random distribution within rotor swept disc |
| Reverse direction, *α* (°) | Uniform (min = 20; max = 30) | [6] |
| Brassy trevally burst speed, *v_b_* (BL s^-1^) | Triangular (min = 5; peak = 7; max = 10) | Field study, [7-9] |
| Sergeant fish burst speed, *v_b_* (BL s^-1^) | Triangular (min = 5; peak = 8; max = 10) | Field study, [7-9] |
| Brassy trevally body length, *L_f_* (m) | Lognormal (µ = 0.373, µ_log_ = -1.048; σ = 0.138, σ_log_ = 0.342 ) | Field study |
| Sergeant fish body length, *L_f_* (m) | Normal (µ = 0.156; σ = 0.019 ) | Field study |
| Time to exhaustion, *t_e_* (s) | Uniform (min = 15; max = 20) | [5,7,8] |

Table S1 References

**1.** Verdant Power (2013) Verdant Power Systems. Available: <http://verdantpower.com/what-technology/>. Accessed 24 September 2014

**2.** Marine Current Turbines (2013) SeaGen S. Available: <http://www.marineturbines.com/SeaGen-Products/SeaGen-S>. Accessed 16 March 2014.

**3.** Glass CW, Wardle CS (1989) Comparison of the reactions of fish to a trawl gear, at high and low light intensities. Fisheries Research 7: 249-266.

**4.** Mazur M, Beauchamp D (2003) A comparison of Visual Prey Detection Among Species of Piscivorous Salmonids: Effects of Light and Low Turbidities. Environmental Biology of Fishes 67: 397-405.

**5.** Domenici P, Blake R (1997) The kinematics and performance of fish fast-start swimming. Journal of Experimental Biology 200: 1165-1178.

**6.** Wardle CS (1986) Fish behaviour and fishing gear. In: Pitcher T, editor. The behaviour of teleost fishes. London: Croom Helm. pp. 469-495.

**7.** Hammer C (1995) Fatigue and exercise tests with fish. Comparative Biochemistry and Physiology Part A: Physiology 112: 1-20.

**8.** Bainbridge R (1960) Speed and Stamina in Three Fish. Journal of Experimental Biology 37: 129-153.

**9.** Videler JJ, Wardle CS (1991) Fish swimming stride by stride: speed limits and endurance. Reviews in Fish Biology and Fisheries 1: 23-40.
